# Clinical efficacy and safety of the Jetstream Atherectomy System in Chinese Patients with femoropopliteal artery occlusive disease

**DOI:** 10.1186/s42155-026-00734-3

**Published:** 2026-08-19

**Authors:** Xin Jia, Wei Guo, Zhong Chen, Xiaoming Zhang, Weiguo Fu, Xinwu Lu, Hongkun Zhang

**Affiliations:** 1 Vascular Surgery Department, Chinese PLA General Hospital, Beijing, China; 2 Vascular Surgery Department, Fudan University Affiliated Zhongshan Hospital, Shanghai, China; 3 Vascular Surgery Department, Beijing Anzhen Hospital, Beijing, China; 4 Vascular Surgery Department, Peking University People’s Hospital, Beijing, China; 5 Vascular Surgery Department, Shanghai Jiaotong University Affiliated the 9th Hospital, Shanghai, China; 6 Vascular Surgery Department, Zhejiang University the First Affiliated Hospital, Hangzhou, China

**Keywords:** Atherectomy, Femoropopliteal artery occlusive disease, Calcified lesions

## Abstract

**Purpose:**

This prospective, multicenter, single-arm clinical trial evaluated the safety and efficacy of the Jetstream Atherectomy System in 72 Chinese patients with calcified femoropopliteal artery occlusive disease (FPOAD).

**Materials and methods:**

The study protocol was approved by the institutional review boards of all participating centers, and all participants provided written informed consent. The primary endpoints were 30-day major adverse events (MAE) and changes in angiographic stenosis. Secondary endpoints included vessel patency, clinical improvement, and adverse events during follow-up.

**Results:**

The study cohort had a mean age of 67.5 ± 9.0 years, with a high prevalence of hypertension (79.2%) and diabetes (37.5%). Severe calcification (PACSS, Peripheral Arterial Calcium Scoring System, grade 3–4) was present in 43.1% of lesions. Technical success (≤ 30% residual stenosis) was achieved in 91.7% of cases. The 30-day major adverse events (MAE) rate was 1.4%, with one target lesion revascularization (TLR), and no deaths or amputations were recorded. Mean stenosis was reduced from 87.8 to 48.5%, with a mean lumen gain of 40.2%. At 12 months, primary patency was 68.4%; secondary patency was 89.7%. Clinical improvement was evidenced by an increase in the mean ankle-brachial index (ABI) from 0.52 ± 0.15 to 0.76 ± 0.18, and 91.7% (66/72) of patients showed an improvement of at least one Rutherford class after 1 year.

**Conclusions:**

This study confirms the Jetstream system’s clinical value in Chinese FPOAD patients. Its combination of mechanical cutting and aspiration makes it particularly suitable for Asian-specific calcified lesions, achieving favorable clinical outcomes.

## Introduction

Peripheral artery disease (PAD) has emerged as a pressing public health challenge globally, imposing a substantial burden on healthcare systems and patient quality of life [1, 2]. In China, the prevalence of PAD has risen sharply in recent decades, driven by two key demographic and epidemiological trends: an aging population and a surge in metabolic disorders such as type 2 diabetes mellitus and dyslipidemia [3]. Among the various manifestations of PAD, femoropopliteal artery occlusive disease (FPOAD) stands out due to its high incidence of severe arterial calcification—a pathological feature that significantly complicates endovascular treatment. These challenges highlight the need for innovative endovascular technologies that can directly address arterial calcification and improve long-term clinical outcomes.

The JETSTREAM™ Atherectomy System (Boston Scientific, Marlborough, MA, USA) is a mechanical plaque excision device designed to overcome the limitations of traditional endovascular therapies for calcified FPOAD. Prior international studies have demonstrated the safety and efficacy of the JETSTREAM system in treating FPOAD [4–6]. Despite these promising international data, there remains a critical gap in evidence regarding the performance of the JETSTREAM system in Chinese patients with FPOAD.

The Jetstream China study was designed to address this evidence gap—the first prospective, multicenter clinical trial to evaluate the safety and effectiveness of the JETSTREAM system for the endovascular treatment of calcified superficial femoral artery and/or popliteal artery occlusive lesions in Chinese patients.

## Methods

### Study design and ethics

This was a prospective, multicenter, single-arm clinical trial conducted in strict accordance with the ethical principles of the Declaration of Helsinki. The study protocol was approved by the institutional review boards of all participating centers, and all participants provided written informed consent.

### Inclusion criteria

- Rutherford class II–IV lower extremity ischemic symptoms confirmed by radiological examinations
- Lesions located in the superficial femoral or popliteal artery, with a length ≤ 15 cm

A three-tier screening process was implemented: initial screening by attending physicians, blinded review by vascular imaging specialists, and final confirmation by an independent Data and Safety Monitoring Board.

### Primary endpoints

Safety evaluation: recording of major adverse events (MAE) within 30 days post-procedure, including all-cause mortality, unplanned major amputation, and target lesion revascularization (TLR).

Efficacy evaluation: comparison of improvements in vascular diameter stenosis rates before and immediately after atherectomy based on angiography data.

### Secondary endpoints

- Monitoring of primary and secondary patency rates of target vessels via ultrasound follow-up
- Assessment of improvements in Rutherford clinical classification and ankle-brachial index (ABI)
- Documentation of adverse events during follow-up (including all-cause mortality, amputation, and target vessel reintervention)

### Procedural specifications

**Fig. 1:**
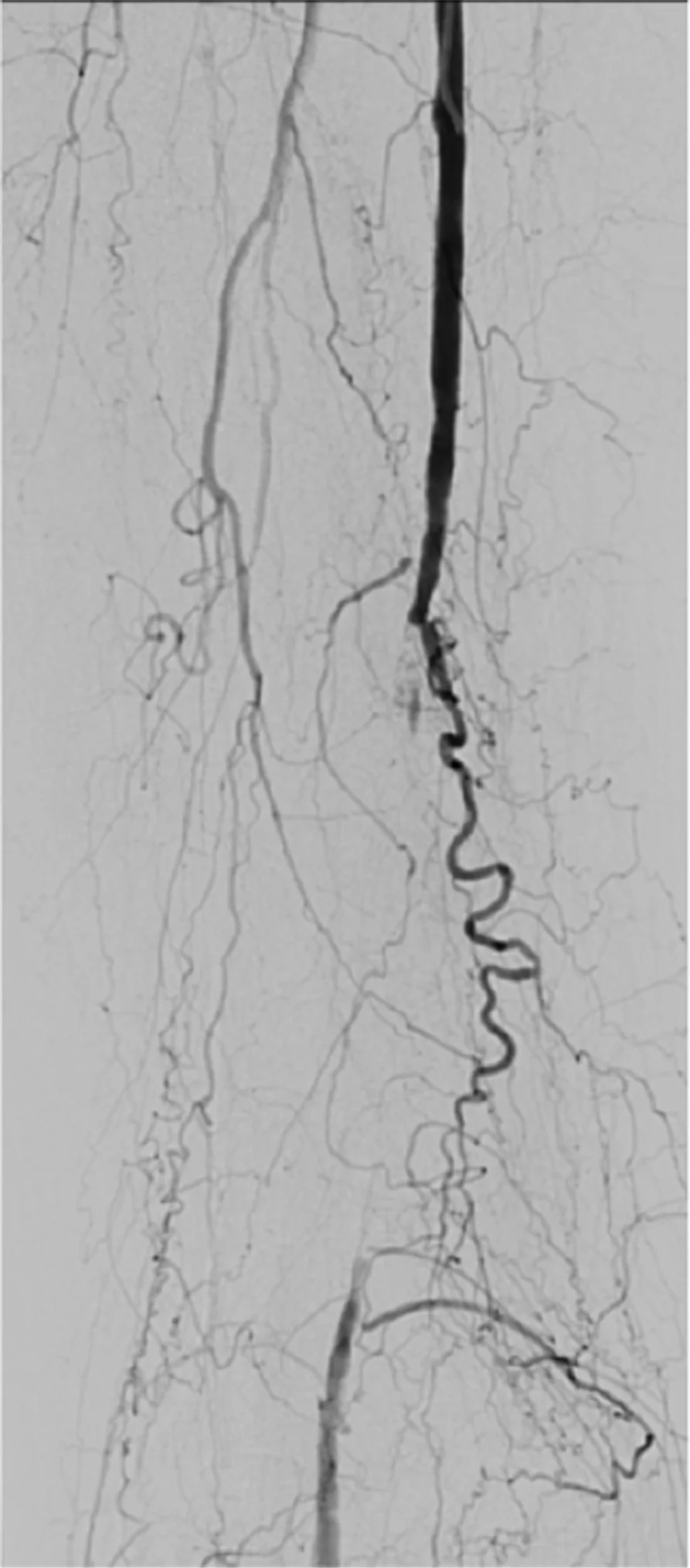
Baseline angiography image

**Fig. 2:**
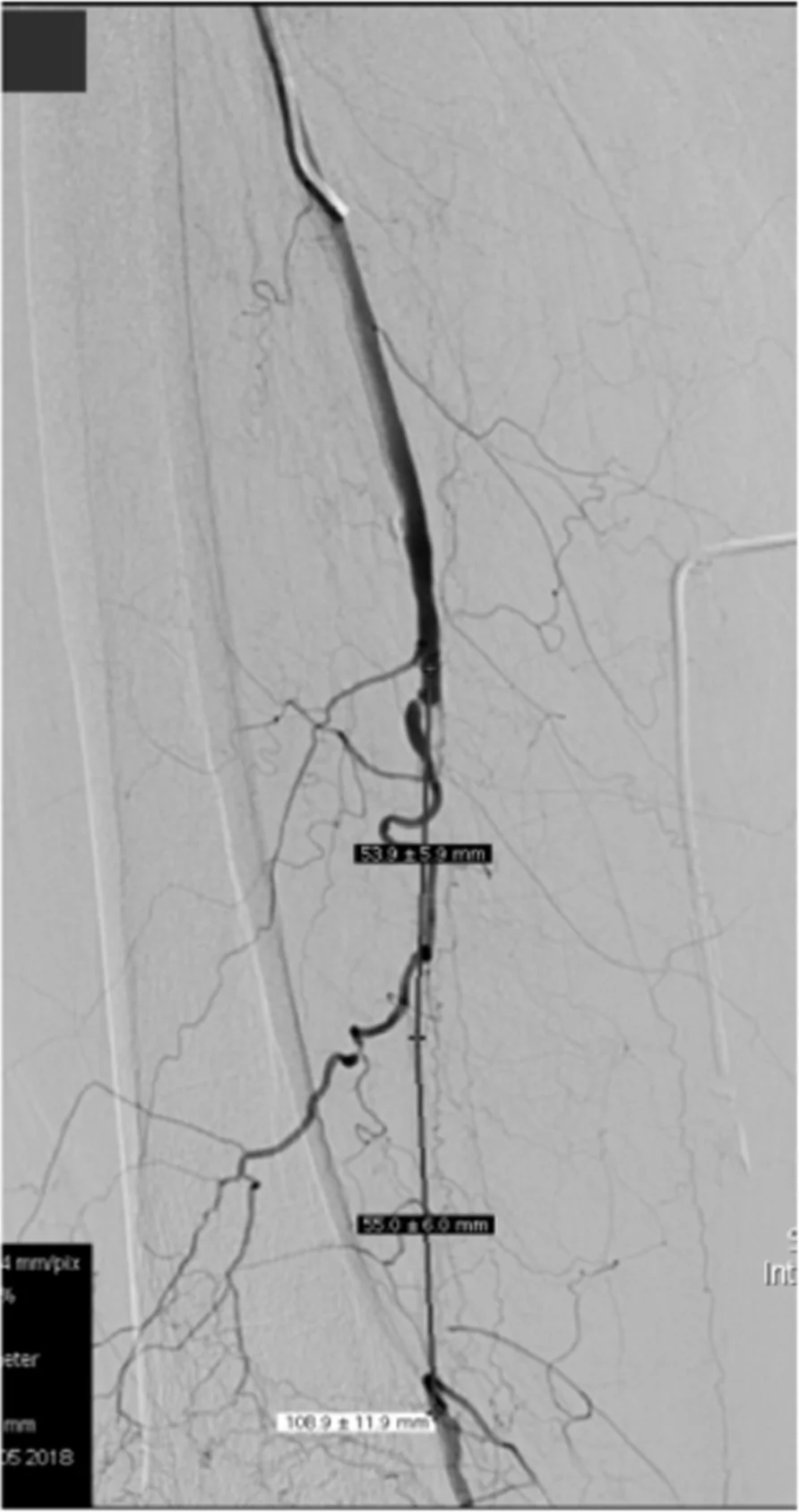
Lesion measurements

**Fig. 3:**
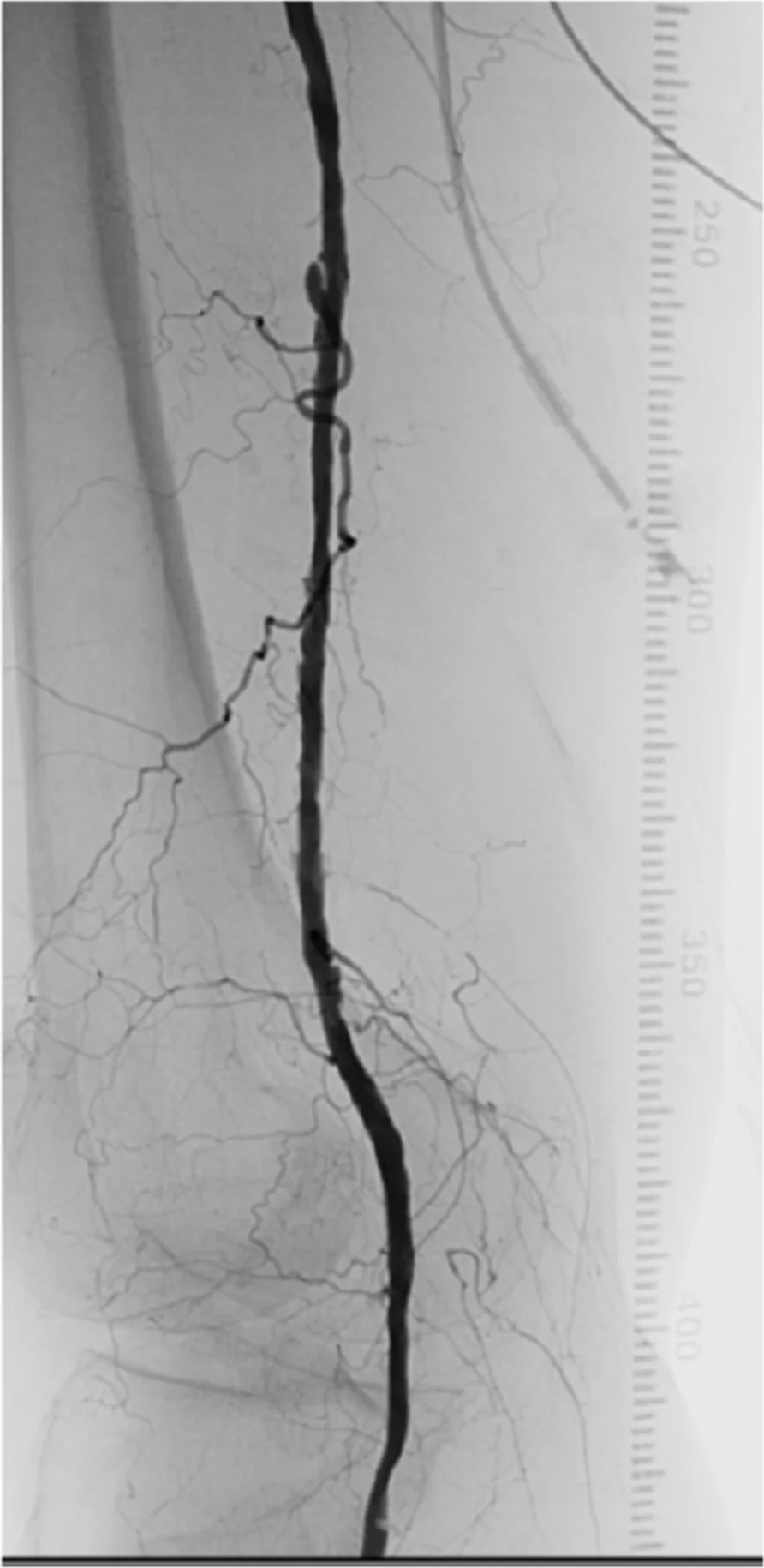
Angiography after atherectomy with Jetstream

**Fig. 4:**
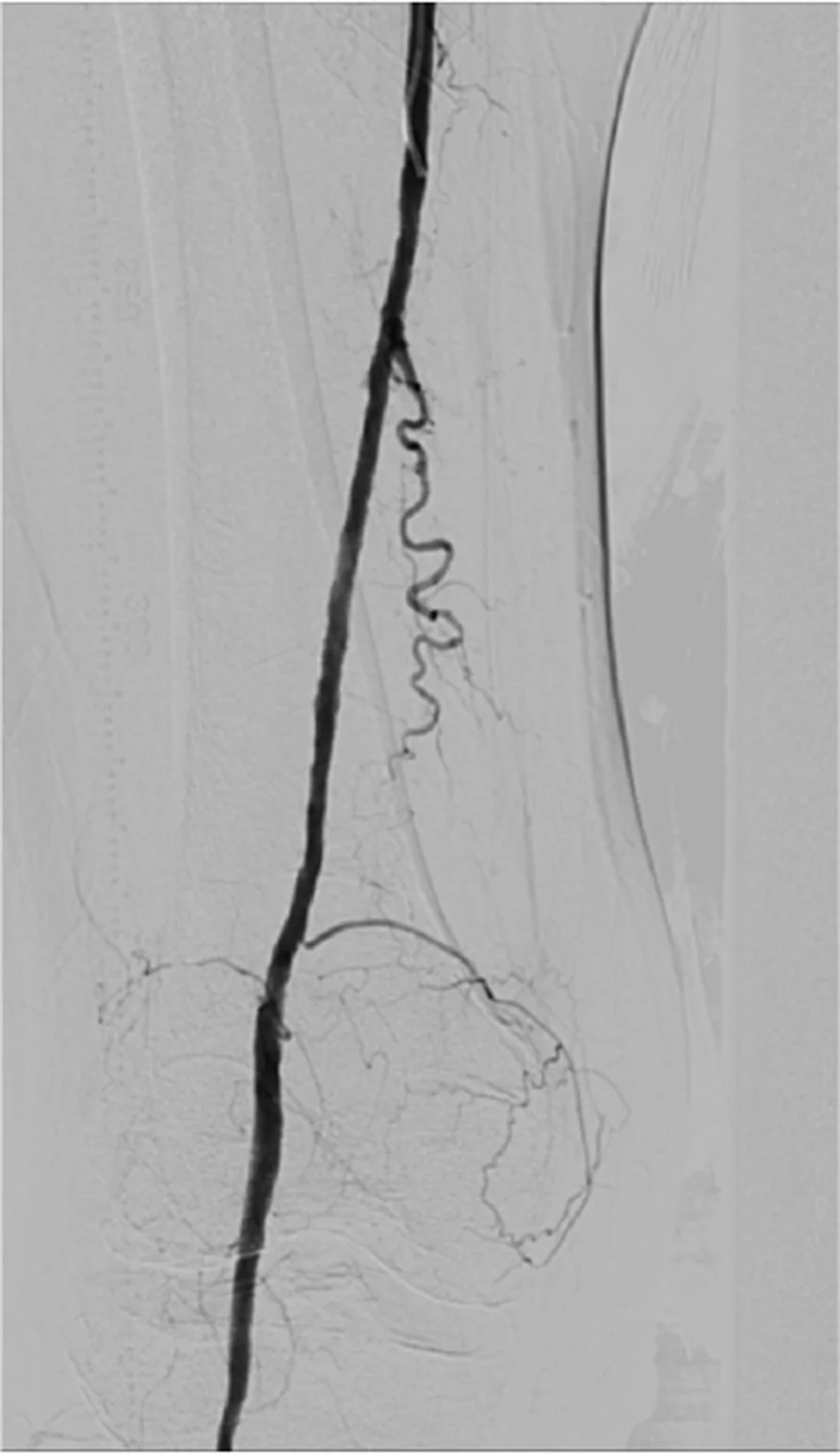
Final angiography image after balloon dilatation

All procedures were performed by certified interventionalists trained in the Jetstream system. The standard protocol included:

- Access via ipsilateral or contralateral femoral artery using a 6 F sheath
- Crossing the lesion with a dedicated guidewire
- Selection of an appropriately sized Jetstream cutting head
- Rotational cutting with simultaneous aspiration
- Post-procedural quantitative angiography for precise assessment of residual stenosis (Figs. 1, 2, 3, and 4)

Technical success was defined as ≤ 30% residual angiographic stenosis in 2 near-orthogonal projections, after successful debulking and adjunctive interventions of the target lesion.

### Statistical methods

Continuous variables were expressed as mean ± standard deviation (SD) and compared using *t*-tests. Categorical variables were analyzed using chi-square tests or Fisher’s exact tests. Kaplan–Meier analysis was used to estimate patency rates.

## Results

### Patient baseline demographics

The study cohort consisted of 72 patients, with a male predominance (83.3%). The mean age was 67.5 ± 9.0 years old.

Comorbidities were distributed as follows: diabetes mellitus in 37.5% (27/72) of patients, hypertension in 79.2% (57/72), and renal insufficiency (eGFR < 60 mL/min/1.73 m^2^) in 15.3% (11/72). These demographic characteristics indicate a population with multiple comorbidities and elevated cardiovascular risk (Table 1).

**Table 1:** Patient baseline demographics

Baseline demographics	
Total number of patients	72
Gender (male)	83.3% (60/72)
Mean age (years)	67.5 ± 9.0
Comorbidities	
Diabetes mellitus	37.5% (27/72)
Hypertension	79.2% (57/72)
Renal insufficiency	15.3% (11/72)

### Lesion characteristics

The mean lesion length was 104.1 ± 61.4 mm. Lesions were localized to the superficial femoral artery in 58.3% (42/72) of patients, involved the popliteal artery in 41.7% (30/72), and were isolated to the popliteal artery in 11.1% (8/72).

Moderate calcification (PACSS grade 2) was observed in 33.3% (24/72) of patients, and severe calcification (grades 3–4) was present in 43.1% (31/72). Thrombus was identified in 8.3% (6/72) of cases.

### Adjuvant interventions post-debulking

Plain balloon angioplasty was performed in 79.2% (57/72) of patients, drug-coated balloon angioplasty in 18.1% (13/72), bailout stent implantation in 11.1% (8/72), and embolic protection devices were used in 47.2% (34/72). The adoption of adjuvant intervention devices was attributed to institutional preferences or reimbursement policies.

In the absence of distal protection devices, the incidence of minor embolization reached 10.5% (4/38); all events were successfully resolved by thrombus aspiration without clinical sequelae.

### Safety outcomes

The 30-day major adverse event (MAE) rate was 1.4% (1/72), consisting of one case of target lesion revascularization (TLR). There were no deaths or unplanned major amputations.

### Efficacy outcomes

Technical success was achieved in 91.7% (66/72) of cases.

Hemodynamic improvements included a reduction in mean stenosis from 87.8 ± 14.4 to 48.5 ± 13.3%, a mean lumen gain of 40.2 ± 15.1%, and an increase in lumen diameter from 1.8 ± 0.6 to 3.2 ± 1.1 mm (Table 2).

**Table 2:** Lesion characteristics and efficacy outcomes

Lesion characteristics	
Mean lesion length (mm)	104.1 ± 61.4
Lesion location	
Superficial femoral artery	58.3% (42/72)
Popliteal artery	41.7% (30/72)
Calcification grade	
Moderate (PACSS grade 2)	33.3% (24/72)
Severe (PACSS grade 3–4)	43.1% (31/72)
Thrombus	8.3% (6/72)
Efficacy outcomes	
Technical success rate	91.7% (66/72)
Mean lumen gain%	40.2 ± 15.1

Notably, the severe calcification subgroup (*n* = 31) exhibited a slightly lower lumen gain (35.6 ± 13.4%), consistent with the technical challenges associated with heavily calcified lesions.

### Clinical follow-up results

Kaplan–Meier-estimated target lesion primary and secondary patency rates were 74.0% (95% CI: 68.2–79.8) and 92.5% (95% CI: 87.3–97.7) at 6 months and 68.4% (95% CI: 62.1–74.7) and 89.7% (95% CI: 83.5–95.9) at 12 months, respectively. The bailout interventions to maintain secondary patency included placement of bare metal stent in 2 cases and drug-coated balloon (DCB) in one case.

Clinical improvement was evidenced by an increase in the mean ankle-brachial index (ABI) from 0.52 ± 0.15 to 0.76 ± 0.18, and 91.7% (66/72) of patients showed an improvement of at least one Rutherford class after 1 year.

## Discussion

The Jetstream China study represents a pivotal step in validating the clinical utility of mechanical atherectomy for Chinese patients with calcified femoropopliteal artery occlusive disease (FPOAD). The results of this prospective, multicenter trial provide compelling evidence that the JETSTREAM™ system is both safe and effective in this patient group, addressing a critical gap in region-specific evidence for endovascular PAD therapies.

A key strength of the Jetstream China study is its consistency with prior international trials of the JETSTREAM system, while also highlighting nuances relevant to Chinese clinical practice [4–6]. The 30-day MAE rate of 1.4% (1 case of target lesion revascularization, no deaths or amputations) is notably low; this may be attributed to several factors: the rigorous three-tier patient screening process in the Chinese study and the specialized training of interventionalists before the study.

In terms of efficacy, the mean lumen gain of 40.2 ± 15.1%—and the fact that this gain was maintained in the severe calcification subgroup (35.6 ± 13.4%)—is particularly noteworthy for Chinese patients, who have a higher prevalence of diabetes-related medial calcification [3]. The JETSTREAM system’s ability to mechanically excise calcific plaque—rather than relying on balloon-mediated expansion—addresses this anatomical challenge, explaining its consistent performance across both patient populations. The JETSTREAM system offers a viable alternative, particularly for patients with moderate-to-severe calcification. The study’s data demonstrate that atherectomy can reduce the need for adjunctive therapy: only 18.1% of patients required drug-coated balloons (DCBs), and 11.1% required bailout stenting. This is significant in China, where bare stent and drug-coated device reimbursement is limited in many regions. By minimizing reliance on these costly adjuncts, the JETSTREAM system can improve cost-effectiveness while maintaining clinical outcomes—a key consideration for China’s public healthcare system.

Despite its strengths, the Jetstream China study has some limitations. The trial was single-arm, meaning there was no control group (e.g., POBA or DCB alone) to directly compare outcomes. Given that DCB access is expanding in China, future studies could evaluate whether this combination strategy further improves outcomes in Chinese patients with severe calcification lesions.

## Conclusion

This study confirms the Jetstream system’s clinical value in Chinese FPOAD patients. Its combination of mechanical cutting and aspiration makes it particularly suitable for Asian-specific calcified lesions, achieving favorable clinical outcomes.

## Data Availability

The data generated and analyzed during the current study are not publicly available due to patient privacy protection and institutional ethical restrictions. Anonymized data may be available from the corresponding author upon reasonable request and with the approval of the institutional review board.
